# 716 Safety and benefits of intraoperative enteral nutrition in critically ill pediatric burn patients

**DOI:** 10.1093/jbcr/irac012.270

**Published:** 2022-03-23

**Authors:** Alexander Morzycki, Alexandra Hudson, Joshua N Wong

**Affiliations:** Division of Plastic and Reconstructive Surgery, University of Alberta, Edmonton, Canada, Edmonton, Alberta; Division of Pediatric Gastroenterology, University of Alberta, Edmonton, Canada, Edmontonn, Alberta; Division of Plastic Surgery, Department of Surgery, University of Alberta, Edmonton, Alberta

## Abstract

**Introduction:**

Burn injuries significantly increase a patient’s metabolic demand. Adequate nutrition is essential to aid in recovery and reduce morbidity and mortality. This is important for pediatric patients, who have limited reserves and are in a period of growth, in contrast to adults. Burn patients often need multiple surgeries, with standard perioperative fasting periods leading to multiple nutritional interruptions. Continuous intraoperative feeding has been a proposed solution, but there is no current consensus on its role and safety, particularly in the pediatric population. The goal of our study was therefore to examine the safety and benefits of intraoperative nutrition in critically ill pediatric burn injury patients.

**Methods:**

A systematic review of *MEDLINE*, *PubMed*, *Scopus*, and *Google Scholar* was conducted using the following terms: F*eeding* or *enteral* or *nutrition* or *fasting* and *adolescent* or *youth* or *pediatric* or *child* or *teen* and *burn* or *thermal injury* or *fire*. The primary outcome was incidence of aspiration. Secondary outcomes included patient nutritional status (caloric deficit, weight), wound healing, days spent in the intensive care unit, ventilator days, pneumonia, number of surgeries, length of hospital stay, and mortality. Pooled analyses of binary outcomes were computed.

**Results:**

Four studies consisting of 496 patients, met inclusion criteria. All studies were level IV evidence, but had high methodological quality. The median burn total body surface area (TBSA) was 43.8% (IQR 33.4-58.8%), with a median of one-third of patients having an inhalational injury. Patients underwent a median of 4.2 surgeries (IQR 1.8-7.4). Intraoperative feeding was conducted through nasoduodenal tubes. There were no aspiration events. Pooled analysis demonstrated that there were no differences in rates of aspiration, pneumonia, or wound infection (p >0.05) between patients who were intraoperatively fed and those who were not. Those fed intraoperatively had significantly more surgeries, ventilator days, longer hospital stays, but lower mortality (p< 0.05). There was large heterogeneity in nutritional assessment methods. Intraoperatively fed patients had an average gain of 144.4 kcal/kg and 1.7 days of exclusive enteral nutrition (vs. loss of -119.1 kcal/kg and -1.4 days) and cumulative positive caloric balance of +2673kcal ±2147 (vs. loss of -7899kcal ±3123), compared to those with interrupted feeding.

**Conclusions:**

Continuous intraoperative duodenal feeding during burn surgery appears to be safe in the Pediatric burn population, with no reported episodes of aspiration. Uninterrupted feeding was also associated with weight maintenance and reduced caloric deficit. It may also have survival benefit, as continuously fed patients needed more surgeries and intensive/hospital care, but had decreased mortality.

